# The Relationship between Household Sanitation and Women’s Experience of Menstrual Hygiene: Findings from a Cross-Sectional Survey in Kaduna State, Nigeria

**DOI:** 10.3390/ijerph15050905

**Published:** 2018-05-03

**Authors:** Julie Hennegan, Linnea Zimmerman, Alexandra K. Shannon, Natalie G. Exum, Funmilola OlaOlorun, Elizabeth Omoluabi, Kellogg J. Schwab

**Affiliations:** 1 The Water Institute, Department of Environmental Health and Engineering, Johns Hopkins Bloomberg School of Public Health, 615 N Wolfe, Baltimore, MD 21205, USA; jhenneg1@jhu.edu (J.H.); nexum1@jhu.edu (N.G.E.); 2 Department of Population, Family and Reproductive Health, Johns Hopkins Bloomberg School of Public Health, 615 N Wolfe, Baltimore, MD 21205, USA; linnea.zimmerman@jhu.edu (L.Z.); ashannon@jhu.edu (A.K.S.); 3 Department of Community Medicine, College of Medicine, University of Ibadan, Queen Elizabeth Road, University College Hospital, Ibadan 200284, Nigeria; fmolaolorun@gmail.com; 4 Center for Research Evaluation Resources and Development, 17 Ajanaku Estate, Ile-Ife 220005, Nigeria; elizomoluabi@gmail.com; 5 Department of Statistics, University of the Western Cape, Robert Sobukwe Rd, Bellville, Cape Town 7535, South Africa

**Keywords:** menstrual hygiene, sanitation, women’s health, menstrual health, cross-sectional survey

## Abstract

Global efforts to improve sanitation have emphasized the needs of women and girls. Managing menstruation is one such need, yet there is scarce research capturing current practices. This study investigated the relationships between household sanitation and women’s experience of menstrual management. Secondary analyses were undertaken on data from 1994 women and girls collected through the Performance Monitoring and Accountability 2020 survey in Kaduna, Nigeria. In multivariable models, women had higher odds of using the main household sanitation facility for menstrual management when they had access to a basic (OR = 1.76 95%CI 1.26–2.46) or limited (OR = 1.63 95%CI 1.08–2.48) sanitation facility, compared to an unimproved facility. Women with no household sanitation facility had higher odds of using their sleeping area (OR = 3.56 95%CI 2.50–5.06) or having no facility for menstrual management (OR = 9.86 95%CI 5.76–16.87) than women with an unimproved sanitation facility. Menstrual management locations were associated with ratings of their characteristics. Safely managed or basic sanitation facilities were not rated more favorably than unimproved facilities in privacy (OR = 1.02 95%CI 0.70–1.48), safety (OR = 1.45 95%CI 0.98–2.15), access to a lock (OR = 0.93 95%CI 0.62–1.37), or soap and water (OR = 1.04 95%CI 0.70–1.56). Women using their sleeping area had more favorable perceptions of their environment. Findings suggest household sanitation influences women’s choices for menstrual management, but that existing indicators for improvement are not sensitive to menstrual needs.

## 1. Introduction

Across contexts, women spend a significant proportion of their lives menstruating. For many, this natural, monthly process presents a challenge to dignity and participation in society. A growing body of studies have drawn attention to the health, education and psychosocial consequences women face due to unmet menstrual needs [1,2,3,4,5]. These needs include access to resources such as clean menstrual materials, soap, water and supportive infrastructure, as well as information including education about menstruation and its management [6,7].

Whilst there is broad acknowledgement of the importance of sanitation facilities and infrastructure as a vital part of menstrual hygiene, limited research has attended to this requirement. Studies that have captured facility needs for menstrual hygiene have focused on girls’ experiences in school environments [8,9]. Qualitative studies in sub-Saharan Africa have noted the stress girls experience in changing their menstrual materials, with many citing safety and privacy concerns as reasons to avoid attending school or changing menstrual absorbents at school [2,10]. The ability to lock doors, and access to soap and water for washing have also been emphasized to support dignity and hygiene [11,12,13]. Limited quantitative studies have attended to the role of facilities for menstrual management. In a systematic review of 138 studies of menstrual hygiene practices in India, only 17 investigated girls changing practices in school facilities, and 21 studies asked if girls had a toilet at home, with only half of girls reporting this to be the case [14]. The authors noted the poor quality of the included studies, most of which failed to engage with the role of girls’ access to infrastructure for menstrual management. A focus on the school environment has led to a focus on sanitation facilities as the primary location for menstrual management activities such as changing materials. Findings to date have contributed to policy and practice recommendations such as ‘girl-friendly’ sanitation facilities which support menstrual hygiene, discussed below. The menstrual practices of adult women, and girls outside of the school environment, have been largely neglected in research and programming [5,15]. Significant stress caused by sanitation insecurity, however, indicates this is likely a significant challenge for both girls and women at home [16,17], with a recent study finding women identified menstruation as their most stressful sanitation activity [18]. More research is needed to understand the intersection between sanitation facilities, other infrastructure, and women and girls’ menstrual management.

In 2015, the Sustainable Development Goals (SDGs) set aspirations and priorities for the development agenda. Menstrual hygiene has been linked to numerous objectives, such as health (SDG3), education (SDG4), and gender equality (SDG5) [19]. SDG6 states an objective to “ensure access to water and sanitation for all”, and includes specific targets to improve access to water, sanitation and hygiene, with Goal 6.2 acknowledging the need to “pay special attention to the needs of women and girls” [20]. Menstruation represents one such need. However, as noted above, there is limited evidence to understand women’s use of sanitation and other facilities for menstrual management. Moreover, indicators for measuring progress towards SDG6.2 focus on ending open defecation, and hygienic separation of excreta from human contact in “safely managed” facilities. Categorizations of open defecation, unimproved, limited, basic and safely managed facilities as outlined by the Joint Monitoring Program [20] focus on how waste is managed, but do not reflect other features of the environment that may be relevant to the needs of women and girls [21]. In India’s national menstrual hygiene guidelines, sanitation facilities are recommended to support menstrual hygiene by: having separated latrines for males and females for privacy; water and soap for cleaning; adequate space in the cubicle for changing and washing; a shelf to keep clothing or menstrual absorbents dry; a mirror for checking stains; and, a clean, functional latrine [22]. Notably, only the final one of these features fits with current benchmarks for sanitation facilities which focus on the management of human excreta [20].

Loughnan and colleagues [23] have noted the importance of indicators capturing menstrual hygiene in raising awareness to the issue, informing policy, and identifying sustainable options for improvement. Of particular interest to the water, sanitation and hygiene (WASH) sector, which has taken leadership on menstrual hygiene [24], is the link between current SDG6 monitoring and improvements in menstrual health. As such, Loughnan and colleagues proposed applying existing WASH indicators as proxies for measuring inadequate menstrual hygiene [23]. In consultation with experts they identified open defecation, unimproved sanitation facilities, lacking access to soap and water for handwashing, and a lack of piped water to the household as proxy indicators of inadequate menstrual hygiene. In a study leveraging Demographic and Health Surveys (DHS) and Multiple Indicator Cluster Surveys (MICS) they estimated access to menstrual hygiene in ten test countries. While these estimates may provide some indication of access, it is unclear how well current indicators capture menstrual hygiene. First, women may use a variety of locations beyond the sanitation facility for menstrual management. This may occur out of necessity as they lack access to a facility, or because other locations are preferred and offer greater privacy, safety and cleanliness. Second, taboos around menstruation may mean that even if women have access to sanitation and handwashing facilities, these may not be designed to support menstrual management. Focus on exiting indicators for improved sanitation facilities may improve access to menstrual hygiene, however, this myopic approach may result in misguided policy that is not sensitive to the needs of women and girls if these standards are unrelated to women’s dignity and stress in managing menstruation. Thus, there is an urgent need for more research to determine the appropriateness of current proxies and improve understanding of women’s use of sanitation and other infrastructure for menstrual management.

### The Present Study

The present study uses Performance Monitoring and Accountability 2020 (PMA2020) data. PMA2020 is a multi-year program undertaking rapid-turnaround surveys to monitor family planning, WASH, and menstrual hygiene across 11 countries. The project uses resident enumerators and a strong network of in-country partner institutions to undertake multiple waves of surveys and produce publicly available data sets [25]. This study uses data from PMA2020 data collected in Kaduna state, Nigeria to provide the first analysis of the interaction between household sanitation and women and girls’ menstrual hygiene practices. First, we evaluated the association between having access to different types of sanitation facilities in the household (safely managed or basic, limited, unimproved, or open defecation) and women and girls’ self-reported location of menstrual management including; the main household sanitation facility, another sanitation facility, their sleeping area, or no facility (i.e., the backyard or field). Second, we compared women’s perceptions of the characteristics of their menstrual management environment, including cleanliness, privacy, safety, the presence of a lock, and access to soap and water, according to the type of location they used. These analyses were adjusted for socio-demographic characteristics, the type of menstrual material used, and the presence of a handwashing facility in the household, which were hypothesized to be associated with the quality of sanitation facilities, women’s choice, and preferences for management locations.

By investigating these research questions, this study provides insights into the usefulness of existing sanitation indicators for estimating access to menstrual hygiene, women’s selection of location for menstrual management, and perceptions of different environments for menstrual management. Further, we provide a brief analysis of women’s response patterns to ratings of their menstrual management location to provide feedback on current outcome assessment such as the consistency of reporting across interrelated concepts of privacy, safety, and facility lockability. Findings are discussed considering the limitations of secondary data analysis, and with attention to policy implications.

## 2. Materials and Methods

### 2.1. Design and Participants

This study presents secondary analyses of data from the PMA2020 survey program in Kaduna state, Nigeria collected between August and September 2015 [26]. The survey employed a two-stage cluster design in which 66 enumeration areas in Kaduna were drawn from the National Population Commission’s master sampling frame. Households were mapped in each enumeration area and 35 were randomly selected and surveyed. All females in surveyed households aged 15 to 49 were asked to participate in the female questionnaire. The final sample included 2934 females.

### 2.2. Study Context

Kaduna, considered as the industrial center of Northern Nigeria, is home to over 60 ethnic groups and well known for its textiles and pottery, among other products. According to the Nigerian 2006 Housing and Population Census, Kaduna’s population was 6,113,503, with a growth rate of 2.47% [27]. The State’s Sustainable Development Goals Report noted that the proportion of Kaduna’s population using safely managed water sources rose from 52.4% to 65.5% between 2015 and 2017. The state is investing in improving waste disposal and water supply, as well as in constructing toilets, and has established over 1000 community-led WASH committees to support the reduction of open defecation, all in a bid to support the attainment of SDG6 [27,28].

### 2.3. Survey Measures

Both household and female questionnaires are publicly available on the PMA2020 website (https://www.pma2020.org/questionnaires).

#### 2.3.1. Household Questionnaire

Household surveys included items regarding asset ownership, regular household membership, water sources, sanitation, and handwashing facilities. For the present study, household questionnaire items of interest included: household wealth quintile, urban or rural location, the main sanitation facility used by the household, and the presence of a handwashing facility. The household wealth quintile was re-calculated for this study to exclude the type of sanitation facility and water source as these are variables of interest in this study. As such, wealth quintiles differ from those provided in the PMA2020 data set and reflect household assets and building materials.

*Main sanitation facility*. To capture the main household sanitation facility, respondents were asked “What is the main toilet facility used by members of your household?”, with a range of response options including “flush/pour toilets (connected to: piped sewer, septic tank, pit latrine, elsewhere)”, “ventilated improved pit latrine”, “pit latrine with a slab”, “pit latrine without a slap/open pit”, “composting toilet”, “bucket toilet”, “hanging toilet/hanging latrine”, or “no facility/bush/field”. Enumerators received training to assist in the correct identification of the sanitation facility. Sanitation facilities were grouped into four categorizations: (1) safely managed or basic facility, i.e., improved sanitation facilities not shared with other households (e.g., flush toilet to sewer or septic tank, pit latrine with slab), (2) limited sanitation facilities, i.e., improved sanitation facilities that were shared with other households, (3) unimproved sanitation facilities (e.g., pit latrine without slab, bucket toilet), and (4) open defecation (i.e., no sanitation facilities). Categories were defined according to updated Joint Monitoring Program ladders [20] for sanitation facilities, with guidance in identifying improved and unimproved facilities from the 2013 Nigeria Demographic and Health Survey [29].

#### 2.3.2. Female Questionnaire

Female surveys included items on contraceptive use and access, socio-demographics, and menstrual hygiene management. Sociodemographics in the present study included marital status, grouped into “never married”, “divorced/widowed”, and “married or cohabitating”, age, and the highest level of education completed.

*Menstrual management location.* Women were asked “What was the main place you used for changing your used pads, cloths, or other sanitary materials?” with response options: “Main household sanitation facility”, “other household sanitation facility”, “sanitation facilities at school”, “sanitation facilities at work”, “other public sanitation facility”, “sleeping area”, “backyard”, or “no facility/bush/field”. For the present study, these were grouped into four categories: main household sanitation facility, other sanitation facility (including other sanitation facilities in the home, at school, work and public sanitation), sleeping area, and backyard/no facility. It should be noted that there were no response options to capture other locations such as bathing or laundry areas, and these locations may have been captured under those recorded as using “other household sanitation facilities”.

For the second study objective, the type of “main household sanitation facility” was integrated to further categorize the menstrual management location. Those using their “main household sanitation facility” for menstrual management were divided into sub-groups according to the type of facility reported in the household questionnaire, as described above. This resulted in 6 categories of menstrual management location: (1) safely managed or basic facility; (2) limited facility; (3) unimproved facility; (4) other sanitation facility; (5) sleeping area; and (6) backyard/no facility. It was not possible to ascertain the type of sanitation facility for those reporting use of an “other sanitation facility” as only the main household sanitation facility type was collected in the household questionnaire.

*Management location characteristics*. Women were asked to report on their menstrual management location characteristics through the item: “While managing your menstrual hygiene, was this place…” followed by a list of: clean, private, safe, able to be locked, supplied with clean water, supplied with soap. Women responded “yes” or “no” to each item. Due to high concordance of responses to the presence of water and soap, these items were combined.

*Menstrual management materials.* Women were asked to report on all the materials they used to “collect or absorb menstrual blood” during their last menstrual period. Women could select multiple responses. For the present study, independent groupings were formed reflecting women using cloths combined with other materials, sanitary pads combined with other materials, a combination of pads and cloths, and other materials which included cotton wool, toilet paper, foam from mattresses, or an ‘other’ response. In a subsequent question, women were asked if they washed and reused sanitary materials during their last menstrual period, with “yes” or “no” response options.

### 2.4. Analyses

Analyses were undertaken using Stata version 15.1 [30]. Descriptive statistics captured participant characteristics, and cross-tabulations display percentages reflecting the proportion of women using different sanitation facilities according to sociodemographics, or women reporting the presence of characteristics of their menstrual management location. Proportions were adjusted for the complex survey design using weights provided in the PMA2020 data set.

To address the first study objective, univariate logistic regressions present associations between household sanitation and women’s location for menstrual management. Multivariable logistic regressions present associations with adjustment for sociodemographic characteristics, the type of menstrual material used, and the presence of a handwashing facility which were hypothesized to influence the relationships between household sanitation and choices for menstrual management location. Binary outcome variables are dummy variables capturing each of the four locations used for menstrual management in contrast to all other possible locations, for example, use of the main household sanitation facility compared to all other locations, and use of the sleeping area rather than any other location. Whilst this means contrasts are not orthogonal, binary logistic regressions were preferred to multinomial logistic regressions for their interpretability. A dichotomous variable reflecting if menstrual materials were washed and reused was used in multivariable models in preference to a multi-category variable describing the types and combinations of menstrual materials used.

To address the second study objective, single and multivariable logistic regressions are reported. The relationships between the self-reported menstrual management location and women’s assessment of this location were assessed, with adjustment for hypothesized socio-demographic and menstrual management confounds. Binary dependent variables indicated women’s assessment of their management location on five characteristics: privacy, safety, cleanliness, presence of a lock, and access to soap and water. Regression analyses were not weighted for survey sampling. Standard errors were adjusted for clustering at the household level, as multiple women within the household were eligible to complete the female questionnaire and this was deemed a more proximal and critical source of bias.

Additional analyses are presented to explore the associations between women’s reports of their environment. Bivariate correlations assessed the association between each reported characteristic, and Cronbach’s alpha was calculated to test internal consistency were all items to be combined as a scale. As noted in the background, we hypothesized that having a lock on the menstrual management location would be associated with greater reports of safety and privacy and this was explored with a chi-square analysis.

### 2.5. Ethical Approval

Approval for human subjects research was granted by National Health Research Ethics Committee (NHREC) of Nigeria (approval code NHREC/01/01/2007-29th/09/2017B). The informed consent process for all female interviews is administered by PMA2020 resident enumerators. Interviews are conducted face-to-face only with respondents who provide explicit and informed consent to participate; in Nigeria, women are asked to confirm their consent verbally. All female interviews are conducted with auditory privacy and, when possible, visual privacy.

## 3. Results

### 3.1. Participants and Sample Characteristics

The response rate for the household questionnaire was 99.0%, and 97.9% for eligible females [25]. For inclusion in the present study, females were required to have had a menstrual period within the three months prior to the survey (*n* = 2006) and to have valid (non-missing) responses for the primary variable of interest: the main location they changed their menstrual absorbent. This resulted in a final sample of 1994 women and girls from 1439 households.

The study included a broad range of women and girls from Kaduna state. Sample characteristics according to the location used for menstrual management, and in aggregate, are displayed in Table 1. The mean age of the study population was 26.66 (SD 8.96). A total of 27.70% of women used only sanitary pads as their menstrual material, with a further 1.36% using pads combined with other materials, and 8.85% using both cloth and sanitary pads. A total of 56.26% of women used only cloth as their menstrual material, with an additional 3.20% using cloth combined with other items, and a final 2.62% using other materials such as cotton wool, toilet paper or foam. To change their menstrual absorbents, 47.21% of women and girls reported using the main household sanitation facility, of which 42.59% used a basic or safely managed facility, 15.15% used a limited facility, and 42.26% used an unimproved facility. A total 15.85% of women reported using another sanitation facility to change their absorbent, with only 15 of the 337 women in this category reporting using a public, school or work facility. A further 28.78% of women used their sleeping area, and 8.16% of women had no facility or used the backyard to change their menstrual materials.

### 3.2. Relationships between Household Sanitation and Menstrual Management Location

Raw and adjusted relationships between household sanitation facilities and the location used for menstrual management are displayed in Table 2, with adjustments for sociodemographic characteristics, menstrual materials and handwashing. In adjusted analyses, women and girls with access to improved (safely managed/basic) sanitation facilities (OR = 1.76 95%CI 1.26–2.46) or limited facilities (OR = 1.63 95%CI 1.08–2.48) had significantly higher odds of using the main household facility to change their menstrual materials than those with an unimproved sanitation facility. Women with improved (safely managed/basic) sanitation facilities or limited facilities did not have significantly higher odds of using another sanitation facility compared to those with an unimproved facility and had significantly lower odds of using their sleeping area or no facility to change their menstrual materials.

In contrast, women with no household sanitation facility had significantly higher odds of using another sanitation facility (OR = 1.85 95%CI 1.09–3.15) or their sleeping area (OR = 3.57 95%CI 2.50–5.06) for menstrual management than those with an unimproved sanitation facility. Women with no household sanitation also had significantly higher odds of using the backyard or no facility to change their menstrual materials (OR = 9.86 95%CI 5.76–16.87) than those with an unimproved facility.

Full univariate and multivariable model relationships, including variables used for adjustment, are presented in Supplementary Materials 1. Relationships between variables of interest and these hypothesized confounders supported their inclusion in adjusted models and provided insights for interpreting results. Younger women had higher odds of using the household facility (OR = 2.18 95%CI 1.53–3.09), while other sociodemographics, and the type of menstrual materials were not significantly associated with use of the main household facility. Older, more educated, and single (never married) women all had higher odds of using another sanitation facility to change their absorbents. Changing menstrual materials in the sleeping area was not associated with demographic characteristics in multivariable models but was significantly more likely for those using reusable absorbents (OR = 1.59 95%CI 1.16–2.18).

### 3.3. Relationships between Women’s Description of Their Menstrual Management Location

Bivariate correlations between reported characteristics of the menstrual management environment are reported in Table 3. There were significant, yet modest, correlations between all characteristics of the menstrual management location. Items considered together had a modest level of internal consistency (Cronbach’s α = 0.57). Further, we investigated the relationship between the presence of a lock and ratings of privacy and safety, hypothesizing that a lock may ensure higher ratings of these perceptions. Of women who reported that their location was lockable, 85.97% reported that it was private, and 83.42% reported it was safe. Among women and girls that reported their management location was not lockable, 64.08% still reported that their location was private, and 68.79% reported it was safe. Thus, women with a lockable location were significantly more likely to report privacy (χ^2^(1) = 114.85, *p* < 0.001), and safety (χ^2^(1) = 56.68, *p* < 0.001). However, many women and girls reported that their management location was private and safe despite not having a lock. Due to the limited interrelationships between the characteristics of women’s menstrual management location, and unique nature of the characteristics assessed, these were considered individually rather than in aggregate.

### 3.4. Women’s Perceptions of Their Menstrual Management Location

Table 4 presents the proportion of women in the sample who rate their menstrual management location as clean, private, safe, lockable and with access to soap and water according to their location of menstrual management, water access, sociodemographics and menstrual materials. Proportions of women reporting favorable characteristics were high, particularly for cleanliness, privacy and safety. Despite managing menstruation in the open, around half of women with no facility still rated their location as clean, private, and safe.

Limited data were available to explore relationships between household hygiene and menstrual experiences. Descriptive comparisons found that 49.79% of women whose household reported having a place to wash their hands, also reported that they had soap and water access in their menstrual management location, meaning 50.21% reported they did not. Of women in households that did not have a handwashing facility, only 26.90% reported that they had access to soap and water in their menstrual management location.

**Figure 1:**
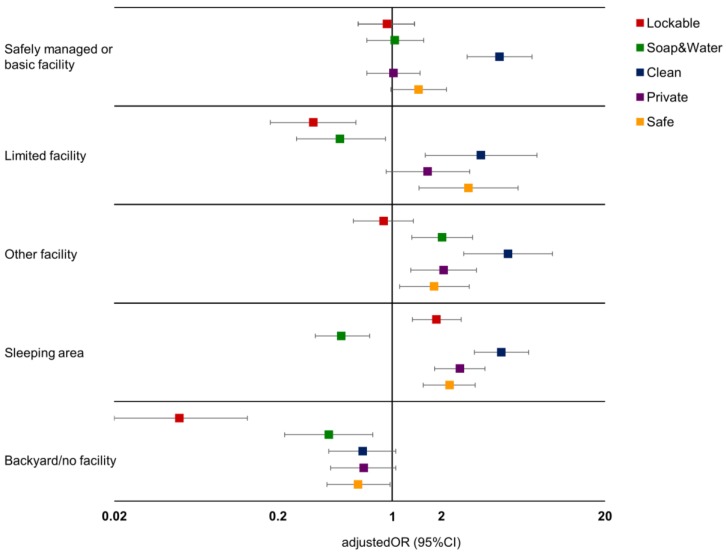
Adjusted odds ratios of the relationship between women’s menstrual management location and their reports of the characteristics of that environment, compared to those using an unimproved sanitation facility (OR = 1.00).

Table 5 presents univariate and multivariable relationships between women’s location for menstrual management and their perceptions of that environment, with adjustments for sociodemographic characteristics, use of reusable menstrual materials, and the presence of a handwashing station in the household. Figure 1 graphically presents the adjusted odds ratios from multivariable regressions in Table 5. There were few statistically significant differences in reported environment characteristics between women using a safely managed/basic facility and those using unimproved sanitation facilities in multivariable models, including if the location was private (OR = 1.02 95%CI 0.70–1.48), safe (OR = 1.45 95%CI 0.98–2.15), lockable (OR = 0.93 95%CI 0.62–1.37) or had access to soap and water (OR = 1.04 95%CI 0.70–1.56), with the exception that safely managed/basic facilities had significantly higher odds of being considered clean (OR = 4.53 95%CI 2.87–7.16). In contrast, women using limited sanitation facilities for menstrual management had higher odds of reporting cleanliness (OR = 3.49 95%CI 1.59–7.67) and safety (OR = 2.93 95%CI 1.46–5.89) than those using an unimproved facility. Limited sanitation facilities had significantly lower odds of being able to be locked (OR = 0.33 95%CI 0.18–0.60) or having access to soap and water (OR = 0.48 95%CI 0.26–0.91) than unimproved facilities. Women using their sleeping area to change their menstrual materials had higher odds of reporting this area could be locked compared to those using an unimproved facility (OR = 1.87 95%CI 1.33–2.64). Models suggested that use of the sleeping area may be particularly beneficial for rural women, leading to follow up analysis for description. For women in a rural area, 57.71% (*n* = 234) reported that their sleeping area was lockable, in contrast to only 33.32% (*n* = 188) reporting a lock was present for those using main household sanitation facilities. Women using their sleeping area also had significantly higher odds of reporting their location was clean (OR = 4.66 95%CI 3.18–6.84), private (OR = 2.60 95%CI 1.82–4.94) and safe (OR = 2.24 95%CI 1.55–3.22), than women using an unimproved facility, but significantly lower odds that this area had access to soap and water (OR = 0.49 95%CI 0.34–0.73). Women using no facility for menstrual management had significantly lower odds of this location being lockable (OR = 0.05 95%CI 0.02–0.13), or having access to soap and water (OR = 0.41 95%CI 0.22–0.76), but were not significantly less likely to report their management location was clean (OR = 0.66 95%CI 0.41–1.05), private (OR = 0.67 95%CI 0.42–1.05), or safe (OR = 0.62 95%CI 0.40–0.97) than women using an unimproved facility.

Supplementary Materials 2 displays full univariate and multivariable relationships between menstrual management location, sociodemographic characteristics, menstrual materials and women’s rating of their menstrual management location. Again, relationships between variables of interest and hypothesized confounders supported their inclusion in adjusted models and provided insights for interpreting results. Markers of higher socioeconomic status such as education and wealth were positively associated with ratings of the environment. Women in the highest wealth quintile had significantly higher odds of reporting a clean (OR = 3.79 95%CI 1.61–8.90), private (OR = 2.00 95%CI 1.18–3.40), lockable (OR = 4.29 95%CI 2.45–7.50) location with access to soap and water (OR = 2.61 95%CI 1.41–4.85) than women in the lowest quintile, although there was no significant difference in reports of safety (OR = 1.15 95%CI 0.61–2.17). Age was not significantly associated with location characteristics in multivariable models. Rural residence predicted lower odds of a clean location (OR = 0.45 95%CI 0.28–0.73), but increased odds of a private (OR = 2.25 95%CI 1.56–3.23) or safe location (OR = 1.93 95%CI 1.34–2.78).

## 4. Discussion

The present study sought to gain an understanding of the relationship between women’s household sanitation facilities and their self-reported experience of menstrual management in Kaduna State, Nigeria. First, we explored the association between access to different types of facility in the household and women’s location for menstrual management. Second, we assessed the relationship between the type of location that women and girls used to change their menstrual materials, and their ratings of the characteristics of that environment.

Women and girls with access to safely managed or basic and limited sanitation facilities had significantly higher odds of changing their menstrual materials in this main household facility compared to those with unimproved sanitation facilities in multivariable models. While this may suggest that improving household sanitation may increase women’s access to such facilities for menstrual management, other relationships suggest that this provides a poor indication of access to an adequate menstrual management location. Women using safely managed or basic sanitation facilities for menstrual management did not provide significantly higher ratings of their locations as private, safe, or lockable, nor were they more likely to report that they had access to soap and water than women using unimproved facilities in multivariable models. Women that managed their menstruation in improved facilities that were shared, limited sanitation facilities, reported higher levels of cleanliness and safety than those using unimproved facilities. It may be that since limited facilities are shared between multiple households there are greater pooled resources available for more significant infrastructure than what is feasible for some at the household level. At the same time, limited facilities were less likely to be lockable or have access to soap and water than unimproved facilities. Taken together, these findings suggest that the presence of a safely managed or basic sanitation facility, as reflected in indicators for SDG6.2, does not mean that women have access to a supportive location for menstrual management. This suggests that current SDG6.2 sanitation indicators may not be sensitive to at least one of the “special needs of women and girls” [20]. In their study of proxies for large-scale assessment of menstrual hygiene, Loughnan et al. [23] suggest unimproved sanitation and open defecation as indicative of inadequate access to menstrual hygiene. Results of this study indicate this distinction is inadequate to capture women’s management location or to consider needs such as privacy and safety.

There is limited existing literature with which to compare the finding that higher levels of sanitation according to existing criteria were not strongly associated with more positive perceptions of the menstrual management environment. However, this finding is consistent with studies of girls’ in schools, which have reported that girls may avoid using sanitation facilities due to stigma around menstruation and fears for privacy [2,12,13], and women’s sanitation-related stress [16,17]. The findings are also consistent with limited, but important, literature which has emphasized that personal hygiene needs beyond sanitation and handwashing; including washing the body and laundry, are critical for women’s wellbeing [31,32,33]. Current indicators for improved sanitation and hygiene also fail to capture such needs.

Approximately 16% of the women and girls surveyed reported that they managed their menstruation in an “other sanitation facility”. For the majority, this was another sanitation facility in their household, with a very small proportion reporting use of a public, school or work facility. This presented a challenge for the present study objectives, as the type of facility that this referred to was unclear. It is likely that this category reflects several different locations. Importantly, the survey item capturing the location of menstrual management included no option for other areas in the household, such as bathing or laundry areas. It is likely that women using these locations for menstrual management were included under this category, and that this does not only capture other sanitation facilities in the household. This is consistent with positive perceptions of the characteristics of this environment, including the presence of soap and water, cleanliness, privacy and safety. It is further supported by associations with sociodemographic factors, which found that use of another facility was positively associated with higher levels of education, and greater wealth.

Many women reported changing their menstrual materials in their sleeping area. This was associated with having access to poorer sanitation facilities (unimproved facilities or open defecation) at the household level. Indeed, over half of women with no household sanitation facility reported changing their absorbent in their sleeping area, with less than one third using no facility. This has significant implications for the use of open defecation at the household level to capture women’s access to a location for menstrual management as proposed by Loughnan and colleagues [23]. Findings of this study suggest that the level of household sanitation does not necessarily indicate women’s menstrual management location or lack of access to a location to change absorbents. Indeed, women rated their sleeping area favorably on many characteristics compared to those using unimproved facilities. Notably, the sleeping area was lockable for a much higher proportion of women in rural areas than the main household sanitation facility. This has important implications for understanding menstrual management practices and education and policy initiatives that may focus solely on the sanitation facility. The finding that age, education, wealth, marital status, and urban residence were not associated with use of the sleeping area may also suggest that this location is not only selected when household sanitation facilities are of poorer quality but may also be preferred by women for other reasons. It should also be noted that women reusing their menstrual materials were more likely to use their sleeping area to change these materials, which may also suggest this location supports storage or washing and drying of absorbents to a greater extent than a sanitation facility. More research is needed to understand women’s preferences for their menstrual management to ensure a women-centered approach to policy and practice. In particular, qualitative research would provide a more nuanced picture of women’s decision making and needs.

Women with no household sanitation facility had much higher odds of having no facility for menstrual management. This was not, however, ubiquitous. Approximately 15% of women who managed their menstruation outside a facility had access to safely managed or basic sanitation facilities, and 22% had unimproved sanitation facilities at home. Contrary to expectations, this was not strongly associated with sociodemographics. This may suggest that unmeasured factors are important. The presence of taboo and stigma around menstruation, or the need to hide menstrual status from others in the household may dictate women changing their menstrual absorbents outside of the house. Women changing menstrual absorbents outside rated this environment the most negatively. Whilst this is consistent with expectations, this study presents the first quantitative support for the burden of a lack of infrastructure for menstrual management on women’s experience of cleanliness, privacy, and safety compared to alternatives.

### 4.1. Measures

The PMA2020 survey program is the first to provide large-scale data on aspects of menstrual hygiene for a broad range of women and girls. As noted above, survey items may require some improvement to capture the variety of locations women and girls’ change their menstrual absorbents. Across characteristics, women reported high rates of cleanliness, privacy and safety in their menstrual management location. Even amongst women who reported having no facility to change their menstrual absorbents, half rated this environment as clean, safe, and private. These findings seem inconsistent with high reports of stress associated with menstrual management in studies amongst school girls in sub-Saharan Africa [2,5,13,34], and among adult women in India [16,18,35]. There are no qualitative studies of the menstrual experiences of adult women in the community in Nigeria against which to compare these ratings, and survey items did not capture other sources of distress such as access to resources needed to manage menstruation, concerns around soiling and odor, or stigma and taboos associated with menstruation identified in existing literature [2,5,8]. Endorsement of the environment as clean, safe, or private may need to be interpreted with caution given the stigmatized nature of menstruation and likely social desirability of positively reporting on the location of management. Further, responses were limited to a dichotomous “yes” or “no” response which is unlikely to capture a nuanced picture of women’s experiences. More research is needed to understand women’s perceptions of their menstrual management, and if dichotomous reports of characteristics (i.e., clean versus not clean) are sufficiently granular. Limited relationships between the characteristics supported use of items individually. While the presence of a lock was associated with increased reports of safety and privacy, many women without access to a lockable door still reported these characteristics. It is unclear if this reflects potential socially desirable responding noted above.

Limited items were available to appraise the adequacy of other menstrual hygiene proxies in current WASH monitoring [23]. The presence of a handwashing station in the household was associated with higher reports of access to soap and water in the location women changed their menstrual materials in descriptive statistics, however, this relationship was not significant in multivariable models. This indicates that the presence of a handwashing facility in the household does not mean this facility is available for menstrual management. At the same time, women were asked about access to soap and water in the location they changed their menstrual absorbents. Responses may be different if women were asked about access to soap and water for washing menstrual absorbents or their body, which may be undertaken in a different location to more frequent absorbent changes.

### 4.2. Strengths and Limitations

The present study utilized large-scale survey data which provided sufficient numbers of women to assess differences across menstrual management locations and captured a broad sample of women across Kaduna state. This represents a different population to much existing menstrual hygiene research which has focused on smaller, rural populations and the experiences of schoolgirls. PMA2020 data was collected in two states in Nigeria, Kaduna and Lagos. Kaduna was selected as the area of interest due to a spread across urban and rural residences, as well as education levels and sanitation facility access. In contrast, Lagos, represents a largely urban population. Similarly, Nigeria was selected as the country of interest for the present study as one of the first sites data were collected on menstrual hygiene in the PMA2020 survey program, and as the population included a range of sanitation facilities and demographic characteristics for comparison. Future research may assess if the findings of this study generalize to other samples in sub-Saharan Africa or low and middle-income contexts. Replications of this study in other available data sets from the PMA2020 survey program may provide further support for reported associations and stronger evidence for the inadequacy of current WASH indicators to capture access to menstrual hygiene. Moreover, more detailed questionnaires designed to improve understanding of women’s needs and level of satisfaction with the facilities they have available for menstrual hygiene would provide insights essential for improving policy and practice.

This study represents an exploratory analysis of the relationship between women and girls’ household sanitation and experiences of menstrual management. The cross-sectional design represents a limitation of the study and precludes causal inference. As a secondary analysis, data were collected prior to the study and survey items were not specifically designed for this research question. Additional items and future studies may further explain women’s choices in their location and their experience of that location, as well as more information on women’s preferences and greater granularity regarding their satisfaction. Limited past research was available to inform hypotheses or to compare the findings of the study. An analytic plan was specified in advance and all conducted analyses are reported here. As noted in the Methods, binary logistic regressions were selected to compare predictors of menstrual management locations. This meant that contrasts were not orthogonal. Increased familywise error from multiple contrasts is likely and effect sizes should be interpreted alongside the confidence interval and the limitations of this design. Given the exploratory nature of the study, these comparisons were preferred to multinominal logistic regressions which would have incorporated all location options in one dependent variable but had more limited interpretability. Differing group sizes for comparison also meant that statistical power varied between these analyses and comparisons of the strength of associations across groups should be interpreted in light of the sample size.

## 5. Conclusions

This study was the first to provide a quantitative evaluation of the relationship between women’s access to sanitation at home and their experience of menstrual hygiene. Results indicate that household sanitation facilities are associated with women’s choice of menstrual management location. Women with access to safely managed or basic facilities had higher odds of changing their menstrual materials in this facility. Women with poorer access to sanitation facilities often used their sleeping area for menstrual management or lacked access to a facility for menstrual management entirely and changed their absorbents outdoors. At the same time, use of a safely managed or basic sanitation facility was not associated with more favorable ratings of the environment when compared to women using unimproved facilities, suggesting that current categorizations and indicators are not sensitive to the menstrual needs of women and girls. In contrast, women using their sleeping area for menstrual management had higher odds of indicating their management location was lockable, clean, private, and safe. Women without access to infrastructure for menstrual management were the most negative about the characteristics of their management location.

## Figures and Tables

**Table 1:** Sample characteristics according to the location of menstrual management.

	Main Household Facility	Other Sanitation Facility	Sleeping Area	Backyard/No Facility	Total
	(*n* = 962)	(*n* = 337)	(*n* = 521)	(*n* = 174)	(*n* = 1994)
	%	%	%	%	%
**Household sanitation (*n* = 1994)**					
Safely managed/basic	57.81	21.32	17.24	3.63	34.78
Limited	52.47	28.02	18.81	0.70	13.63
Unimproved	59.26	7.13	28.30	5.31	33.67
Open defecation	0.00	12.36	59.65	27.99	17.92
**Handwashing place (*n* = 1991)**					
Yes	52.38	25.99	17.50	6.12	28.92
No	44.94	12.59	33.46	9.02	71.08
**Age (*n* = 1994)**					
15–19	51.15	15.74	25.00	8.11	26.94
20–24	46.59	13.17	30.48	9.76	19.60
25–34	46.28	15.64	30.18	7.90	30.52
35 and older	44.36	18.54	29.91	7.20	22.93
**Education (*n* = 1994)**					
None	57.24	4.70	28.45	9.61	35.68
Primary school	46.33	10.72	34.90	8.05	22.53
Secondary school	38.77	26.02	26.88	8.34	33.11
Higher education	40.53	36.20	21.48	1.79	8.68
**Marital status (*n* = 1991)**					
Married or cohabitating	49.53	12.42	29.81	8.24	73.41
Divorced or widowed	38.95	19.06	35.75	6.24	3.46
Never married	41.15	26.31	24.44	8.09	23.13
**Wealth quintile (*n* = 1994)**					
1 (lowest)	44.85	4.18	39.92	11.05	17.29
2	56.62	5.19	24.87	13.32	20.34
3	48.23	6.89	34.53	10.36	17.71
4	43.84	21.69	31.62	2.85	20.29
5 (highest)	43.88	31.84	18.82	5.46	24.37
**Rurality (*n* = 1994)**					
Urban	49.82	28.02	17.79	4.37	28.10
Rural	46.19	11.09	33.07	9.64	71.90
**Menstrual materials (*n* = 1969)**					
Pads (and other items)	41.62	30.42	21.77	6.19	29.06
Cloth (and other items)	49.83	8.43	32.08	9.66	59.46
Pads & cloth	50.24	15.62	30.02	4.12	8.85
Other	57.14	19.13	17.34	6.39	2.62
**Reuse menstrual materials (*n* = 1988)**					
Yes	51.18	9.39	30.38	9.05	67.80
No	38.76	29.42	25.70	6.13	32.20

**Table 2:** Bivariate and multivariable relationships between household sanitation and menstrual management location.

	**Main Household Facility (*n* = 962)**		**Other Sanitation Facility (*n* = 337)**	
	OR (95%CI)	aOR (95%CI)	OR (95%CI)	aOR (95%CI)
**Household sanitation**				
Safely managed/basic	0.93 (0.71–1.23)	1.76 (1.26–2.46)	2.76 (1.81–4.22)	1.22 (0.76–1.97)
Limited	0.72 (0.51–1.02)	1.63 (1.08–2.48)	4.29 (2.63–6.98)	1.70 (0.97–2.98)
Unimproved	1.00	1.00	1.00	1.00
Open defecation	-	-	1.57 (0.96–2.56)	1.85 (1.09–3.15)
	**Sleeping Area (*n* = 521)**		**Backyard/No Facility (*n* = 174)**	
	OR (95%CI)	aOR (95%CI)	OR (95%CI)	aOR (95%CI)
**Household sanitation**				
Safely managed/basic	0.61 (0.44–0.85)	0.57 (0.39–0.82)	0.63 (0.33–1.19)	0.38 (0.16–0.90)
Limited	0.67 (0.45–1.01)	0.59 (0.36–0.98)	0.09 (0.02–0.37)	0.03 (0.00–0.21)
Unimproved	1.00	1.00	1.00	1.00
Open defecation	3.68 (2.65–5.11)	3.56 (2.50–5.06)	7.26 (4.61–11.44)	9.86 (5.76–16.87)

OR: bivariate odds ratio. 95%CI: 95% Confidence Interval. aOR: odds ratio with adjustment for age, education, marital status, wealth quintile, rural residence, use of reusable menstrual materials and the presence of a handwashing facility in the household. - no observations and category excluded from analyses.

**Table 3:** Interrelationships between women’s ratings of the characteristics of their menstrual management location.

	Clean	Private	Safe	Lockable	Soap & Water
**Clean**	1.00				
**Private**	0.24 ***	1.00			
**Safe**	0.38 ***	0.31 ***	1.00		
**Lockable**	0.17 ***	0.24 ***	0.16 ***	1.00	
**Soap & Water**	0.12 ***	0.11 ***	0.01	0.37 ***	1.00

*** *p* < 0.001.

**Table 4:** Proportion of women describing their menstrual management location as clean, private, safe, lockable and having access to soap and water, according to household sanitation, water, and sociodemographics.

	Clean	Private	Safe	Lockable	Soap & Water
	%	%	%	%	%
**Menstrual management location**					
Safely managed/basic	91.26	72.82	80.64	47.30	39.39
Limited	92.19	83.06	90.43	41.44	40.52
Unimproved	56.62	52.99	56.28	26.17	30.50
Other sanitation facility	91.43	86.60	86.82	60.10	62.64
Sleeping area	87.86	81.12	75.52	54.93	18.19
No facility	47.05	45.25	52.49	6.27	19.76
**Handwashing place**					
Yes	91.06	81.95	89.08	56.69	49.79
No	75.36	67.84	66.70	38.00	26.90
**Age**					
15–19	79.42	69.82	71.99	46.53	35.49
20–24	75.05	69.35	73.04	40.36	29.24
25–34	81.47	73.73	71.97	43.18	32.02
35+	82.38	74.13	76.20	43.22	37.22
**Education**					
None	69.84	58.39	64.21	24.34	23.47
Primary school	80.01	72.99	66.77	40.31	27.45
Secondary school	85.88	80.36	82.39	58.89	38.83
Higher education	97.86	92.58	91.40	72.43	71.33
**Marital status**					
Married/cohabitating	77.78	69.20	71.56	38.81	31.32
Divorced/widowed	74.62	81.49	74.57	54.66	29.49
Never married	87.22	79.26	77.91	56.96	41.61
**Wealth**					
1 (lowest)	68.17	55.58	69.44	24.97	19.62
2	68.47	55.46	55.84	18.96	22.98
3	70.99	68.20	65.94	36.49	26.53
4	85.58	80.21	74.28	50.61	32.20
5 (highest)	96.54	89.22	91.78	71.20	55.30
**Rurality**					
Urban	95.21	89.66	88.45	40.04	48.54
Rural	73.88	64.98	67.19	52.49	27.76
**Menstrual materials**					
Pads (+other)	96.24	85.53	88.82	63.05	48.19
Cloth (+other)	70.73	64.92	66.28	31.76	25.91
Pads & cloth	91.88	77.63	77.21	52.01	38.89
Other	70.44	65.91	50.20	64.48	34.80
**Reuse menstrual material**					
Yes	74.79	66.60	68.85	32.63	27.93
No	91.00	83.49	82.49	66.73	45.85

**Table 5:** Univariate and multivariable relationships between sanitation, hygiene, menstrual materials and sociodemographic characteristics and women’s reports of the characteristics of their menstrual management location.

	**Clean**		**Private**		**Safe**	
	OR (95%CI)	aOR (95%CI)	OR (95%CI)	aOR (95%CI)	OR (95%CI)	aOR (95%CI)
**Menstrual management location**						
Safely managed/basic	7.72 (4.98–11.98)	4.53 (2.87–7.16)	2.07 (1.47–2.93)	1.02 (0.70–1.48)	2.18 (1.52–3.11)	1.45 (0.98–2.15)
Limited	11.48 (5.45–24.20)	3.49 (1.59–7.67)	6.09 (3.50–10.62)	1.65 (0.92–2.98)	7.16 (3.77–13.60)	2.93 (1.46–5.89)
Unimproved	1.00	1.00	1.00	1.00	1.00	1.00
Other sanitation facility	11.18 (6.42–19.46)	5.12 (2.74–9.56)	5.89 (3.88–8.91)	2.07 (1.30–3.28)	3.53 (2.30–5.42)	1.81 (1.11–2.96)
Sleeping area	5.47 (3.79–7.90)	4.66 (3.18–6.84)	3.53 (2.53–4.94)	2.60 (1.82–3.69)	2.58 (1.83–3.63)	2.24 (1.55–3.22)
No facility/field	0.84 (0.55–1.28)	0.66 (0.41–1.05)	0.93 (0.62–1.41)	0.67 (0.42–1.05)	0.77 (0.50–1.17)	0.62 (0.40–0.97)
	**Lockable**		**Soap & Water**			
	OR (95%CI)	aOR (95%CI)	OR (95%CI)	aOR (95%CI)		
**Menstrual management location**						
Safely managed/basic	2.01 (1.40–2.88)	0.93 (0.62–1.37)	1.86 (1.27–2.72)	1.04 (0.70–1.56)		
Limited	1.40 (0.90–2.16)	0.33 (0.18–0.60)	1.32 (0.83–2.11)	0.48 (0.26–0.91)		
Unimproved	1.00	1.00	1.00	1.00		
Other sanitation facility	3.21 (2.21–4.66)	0.89 (0.58–1.35)	4.55 (3.07–6.72)	2.02 (1.32–3.10)		
Sleeping area	2.56 (1.85–3.52)	1.87 (1.33–2.64)	0.67 (0.45–0.98)	0.49 (0.34–0.73)		
No facility/field	0.13 (0.06–0.30)	0.05 (0.02–0.13)	0.55 (0.32–0.93)	0.41 (0.22–0.76)		

OR: bivariate odds ratio. 95%CI: 95% Confidence Interval. aOR: odds ratio with adjustment for age, education, marital status, wealth quintile, rural residence, use of reusable menstrual materials and the presence of a handwashing facility in the household.

## Supplementary Materials

The following are available online at http://www.mdpi.com/1660-4601/15/5/905/s1. S1. Bivariate and multivariable relationships between household sanitation, hygiene, menstrual material, demographics and menstrual management location; S2. Univariate and multivariable relationships between sanitation, hygiene, menstrual materials and sociodemographic characteristics and women’s reports of the characteristics of their menstrual management location; S3. STROBE checklist for reporting of observational studies.

## Statement

Data is publicly available by request from the PMA2020 website: https://www.pma2020.org/request-access-to-datasets.
