# Supplementary material for: The Relationship between Household Sanitation and Women’s Experience of Menstrual Hygiene: Findings from a Cross-Sectional Survey in Kaduna State, Nigeria
Source: Int J Environ Res Public Health. 2018 May 3;15(5):905. doi: 10.3390/ijerph15050905 (PMC5981944; doi:10.3390/ijerph15050905)
Supplement: Supplementary file 1 [file ijerph-15-00905-s001.zip › Supplementary materials/Supplementary Materials 1.pdf]

Supplementary Materials 1.

Bivariate and multivariable relationships between household sanitation, hygiene, menstrual material, demographics and menstrual management location

	Main household facility (n=962)		Other sanitation facility (n=337)		Sleeping area (n=521)		Backyard/no facility (n=174)	
	OR (95%CI)	aOR (95%CI)	OR (95%CI)	aOR (95%CI)	OR (95%CI)	aOR (95%CI)	OR (95%CI)	aOR (95%CI)
**Household sanitation**								
Safely managed/basic	0.93 (0.71-1.23)	1.76 (1.26-2.46)	2.76 (1.81-4.22)	1.22 (0.76-1.97)	0.61 (0.44-0.85)	0.57 (0.39-0.82)	0.63 (0.33-1.19)	0.38 (0.16-0.90)
Limited	0.72 (0.51-1.02)	1.63 (1.08-2.48)	4.29 (2.63-6.98)	1.70 (0.97-2.98)	0.67 (0.45-1.01)	0.59 (0.36-0.98)	0.09 (0.02-0.37)	0.03 (0.00-0.21)
Unimproved	1.00	1.00	1.00	1.00	1.00	1.00	1.00	1.00
Open defecation	-	-	1.57 (0.96-2.56)	1.85 (1.09-3.15)	3.68 (2.65-5.11)	3.56 (2.50-5.06)	7.26 (4.61-11.44)	9.86 (5.76-16.87)
**Handwashing facility**								
Yes	1.18 (0.93-1.50)	1.07 (0.79-1.45)	1.91 (1.39-2.63)	1.04 (0.71-1.52)	0.58 (0.44-0.77)	0.84 (0.60-1.19)	0.58 (0.35-0.93)	1.41 (0.84-2.38)
No	1.00	1.00	1.00	1.00	1.00	1.00	1.00	1.00
**Age**								
15-19	1.31 (1.04-1.65)	2.18 (1.53-3.09)	0.88 (0.66-1.17)	0.48 (0.31-0.74)	0.78 (0.59-1.03)	0.70 (0.49-1.01)	0.95 (0.60-1.49)	0.70 (0.39-1.26)
20-24	0.99 (0.75-1.29)	1.45 (1.04-2.03)	0.83 (0.58-1.18)	0.59 (0.38-0.91)	1.04 (0.76-1.41)	0.88 (0.62-1.25)	1.32 (0.81-2.13)	0.91 (0.52-1.56)
25-34	0.97 (0.76-1.25)	1.22 (0.92-1.63)	0.88 (0.64-1.21)	0.76 (0.53-1.10)	1.08 (0.82-1.42)	0.96 (0.71-1.29)	1.15 (0.73-1.79)	0.84 (0.50-1.40)
35+	1.00	1.00	1.00	1.00	1.00	1.00	1.00	1.00
**Education**								
None	1.00	1.00	1.00	1.00	1.00	1.00	1.00	1.00
Primary school	0.58 (0.45-0.75)	0.82 (0.59-1.13)	2.20 (1.43-3.41)	1.59 (1.02-2.49)	1.37 (1.04-1.80)	1.23 (0.91-1.67)	1.06 (0.68-1.66)	0.69 (0.41-1.15)
Secondary school	0.45 (0.35-0.56)	0.68 (0.47-0.99)	5.12 (3.51-7.49)	2.02 (1.28-3.20)	0.98 (0.75-1.27)	1.15 (0.80-1.64)	1.16 (0.77-1.73)	0.94 (0.53-1.64)
Higher education	0.62 (0.42-0.93)	0.88 (0.54-1.42)	9.96 (6.00-16.54)	2.42 (1.37-4.27)	0.42 (0.25-0.70)	0.78 (0.44-1.38)	0.06 (0.01-0.44)	0.08 (0.01-0.66)
**Marital status**								
Married/cohabitating	1.00	1.00	1.00	1.00	1.00	1.00	1.00	1.00
Divorced/widowed	0.90 (0.56-1.44)	1.06 (0.61-1.85)	1.50 (0.78-2.89)	0.96 (0.43-2.12)	1.05 (0.63-1.77)	1.12 (0.63-1.97)	0.57 (0.20-1.58)	0.65 (0.24-1.76)
Never married	0.75 (0.60-0.95)	0.72 (0.51-1.02)	2.41 (1.84-3.15)	1.64 (1.11-2.39)	0.75 (0.57-0.98)	1.03 (0.70-1.50)	0.84 (0.54-1.31)	1.01 (0.55-1.83)
**Wealth**								
1 (lowest)	1.00	1.00	1.00	1.00	1.00	1.00	1.00	1.00
2	1.37 (0.94-2.01)	1.44 (0.87-2.41)	0.82 (0.36-1.87)	0.71 (0.31-1.64)	0.67 (0.45-1.01)	0.68 (0.44-1.04)	1.16 (0.65-2.04)	1.38 (0.76-2.52)
3	1.05 (0.72-1.54)	1.12 (0.67-1.88)	1.20 (0.53-2.67)	0.84 (0.36-1.97)	0.84 (0.57-1.26)	0.86 (0.56-1.31)	1.13 (0.64-1.99)	1.23 (0.66-2.29)
4	0.81 (0.56-1.18)	0.49 (0.82-1.63)	4.00 (1.93-8.29)	2.24 (0.93-5.37)	0.87 (0.58-1.28)	1.39 (0.87-2.21)	0.34 (0.15-0.78)	0.64 (0.26-1.57)
5 (highest)	0.97 (0.67-1.39)	0.50 (0.27-0.95)	6.48 (3.16-13.28)	2.58 (0.91-7.32)	0.35 (0.23-0.53)	0.81 (0.44-1.48)	0.50 (0.27-0.92)	3.62 (1.52-8.64)
**Rurality**								
Urban	1.00	1.00	1.00	1.00	1.00	1.00	1.00	1.00
Rural	0.81 (0.64-1.02)	1.16 (0.82-1.63)	0.41 (0.30-0.56)	1.11 (0.69-1.76)	1.78 (1.36-2.33)	0.72 (0.48-1.09)	3.12 (1.91-5.11)	0.93 (0.47-1.86)
**Reuse menstrual materials**								
Yes	1.34 (1.07-1.67)	1.30 (0.97-1.75)	0.24 (0.18-0.32)	0.46 (0.34-0.64)	2.00 (1.55-2.60)	1.59 (1.16-2.18)	1.26 (0.84-1.88)	0.75 (0.46-1.25)
No	1.00	1.00	1.00	1.00	1.00	1.00	1.00	1.00

OR: bivariate odds ratio. 95%CI: 95% confidence interval. aOR: odds ratio for full multivariable model. - no observations and category excluded from analyses
