# Supplementary material for: The Relationship between Household Sanitation and Women’s Experience of Menstrual Hygiene: Findings from a Cross-Sectional Survey in Kaduna State, Nigeria
Source: Int J Environ Res Public Health. 2018 May 3;15(5):905. doi: 10.3390/ijerph15050905 (PMC5981944; doi:10.3390/ijerph15050905)
Supplement: Supplementary file 1 [file ijerph-15-00905-s001.zip › Supplementary materials/Supplementary Materials 2.pdf]

**Supplementary Materials 2.**

Univariate and multivariable relationships between sanitation, hygiene, menstrual materials and sociodemographic characteristics and women's reports of the characteristics of their menstrual management location

| | Clean | | Private | | Safe | | Lockable | | Soap&Water | |
|---|---|---|---|---|---|---|---|---|---|---|
| | OR (95%CI) | aOR (95%CI) | OR (95%CI) | aOR (95%CI) | OR (95%CI) | aOR (95%CI) | OR (95%CI) | aOR (95%CI) | OR (95%CI) | aOR (95%CI) |
| **Menstrual management location** | | | | | | | | | | |
| Safely managed/basic | 7.72 (4.98-11.98) | 4.53 (2.87-7.16) | 2.07 (1.47-2.93) | 1.02 (0.70-1.48) | 2.18 (1.52-3.11) | 1.45 (0.98-2.15) | 2.01 (1.40-2.88) | 0.93 (0.62-1.37) | 1.86 (1.27-2.72) | 1.04 (0.70-1.56) |
| Limited | 11.48 (5.45-24.20) | 3.49 (1.59-7.67) | 6.09 (3.50-10.62) | 1.65 (0.92-2.98) | 7.16 (3.77-13.60) | 2.93 (1.46-5.89) | 1.40 (0.90-2.16) | 0.33 (0.18-0.60) | 1.32 (0.83-2.11) | 0.48 (0.26-0.91) |
| Unimproved | 1.00 | 1.00 | 1.00 | 1.00 | 1.00 | 1.00 | 1.00 | 1.00 | 1.00 | 1.00 |
| Other sanitation | 11.18 (6.42-19.46) | 5.12 (2.74-9.56) | 5.89 (3.88-8.91) | 2.07 (1.30-3.28) | 3.53 (2.30-5.42) | 1.81 (1.11-2.96) | 3.21 (2.21-4.66) | 0.89 (0.58-1.35) | 4.55 (3.07-6.72) | 2.02 (1.32-3.10) |
| Sleeping | 5.47 (3.79-7.90) | 4.66 (3.18-6.84) | 3.53 (2.53-4.94) | 2.60 (1.82-3.69) | 2.58 (1.83-3.63) | 2.24 (1.55-3.22) | 2.56 (1.85-3.52) | 1.87 (1.33-2.64) | 0.67 (0.45-0.98) | 0.49 (0.34-0.73) |
| No facility/field | 0.84 (0.55-1.28) | 0.66 (0.41-1.05) | 0.93 (0.62-1.41) | 0.67 (0.42-1.05) | 0.77 (0.50-1.17) | 0.62 (0.40-0.97) | 0.13 (0.06-0.30) | 0.05 (0.02-0.13) | 0.55 (0.32-0.93) | 0.41 (0.22-0.76) |
| **Handwashing** | | | | | | | | | | |
| Yes | 4.59 (3.08-6.84) | 2.61 (1.65-4.11) | 2.72 (2.03-3.64) | 1.66 (1.20-2.31) | 4.11 (2.86-5.91) | 2.72 (1.83-4.06) | 2.67 (2.08-3.43) | 2.09 (1.54-2.83) | 2.05 (1.58-2.65) | 1.21 (0.86-1.70) |
| No | 1.00 | 1.00 | 1.00 | 1.00 | 1.00 | 1.00 | 1.00 | 1.00 | 1.00 | 1.00 |
| **Age** | | | | | | | | | | |
| 15-19 | 1.00 | 1.00 | 1.00 | 1.00 | 1.00 | 1.00 | 1.00 | 1.00 | 1.00 | 1.00 |
| 20-24 | 0.81 (0.61-1.08) | 0.84 (0.59-1.21) | 1.01 (0.76-1.33) | 1.17 (0.84-1.63) | 1.08 (0.80-1.45) | 1.07 (0.77-1.49) | 0.83 (0.64-1.09) | 0.86 (0.62-1.19) | 0.81 (0.61-1.09) | 0.83 (0.59-1.16) |
| 25-34 | 1.21 (0.92-1.59) | 1.31 (0.71-1.89) | 1.03 (0.79-1.33) | 1.22 (0.88-1.71) | 1.07 (0.82-1.40) | 1.01 (0.73-1.40) | 0.90 (0.70-1.15) | 1.04 (0.75-1.44) | 0.87 (0.66-1.13) | 0.87 (0.63-1.21) |
| 35+ | 1.35 (1.01-1.82) | 1.37 (0.91-2.08) | 1.29 (0.98-1.69) | 1.44 (1.01-2.06) | 1.25 (0.94-1.66) | 1.12 (0.78-1.62) | 0.83 (0.65-1.06) | 0.88 (0.61-1.25) | 0.94 (0.74-1.21) | 0.94 (0.66-1.34) |
| **Education** | | | | | | | | | | |
| None | 1.00 | 1.00 | 1.00 | 1.00 | 1.00 | 1.00 | 1.00 | 1.00 | 1.00 | 1.00 |
| Primary school | 1.49 (1.12-1.98) | 1.08 (0.77-1.53) | 1.52 (1.16-1.98) | 1.19 (0.88-1.59) | 0.96 (0.73-1.26) | 0.91 (0.67-1.23) | 1.79 (1.37-2.34) | 1.45 (1.06-1.96) | 1.37 (1.01-1.85) | 1.17 (0.83-1.64) |
| Secondary school | 2.55 (1.87-3.46) | 1.25 (0.80-1.94) | 3.16 (2.40-4.16) | 1.70 (1.16-2.47) | 2.35 (1.77-3.12) | 2.04 (1.37-3.03) | 3.25 (2.51-4.19) | 1.81 (1.27-2.57) | 2.29 (1.74-3.00) | 1.28 (0.88-1.88) |
| Higher education | 15.48 (5.33-44.94) | 2.24 (0.75-6.71) | 12.19 (6.06-24.52) | 3.43 (1.61-7.31) | 5.94 (3.14-11.22) | 2.33 (1.14-4.76) | 5.92 (3.87-9.05) | 2.03 (1.20-3.42) | 7.31 (4.74-11.28) | 2.76 (1.64-4.65) |
| **Marital status** | | | | | | | | | | |
| Married/cohabitating | 1.00 | 1.00 | 1.00 | 1.00 | 1.00 | 1.00 | 1.00 | 1.00 | 1.00 | 1.00 |
| Divorced/widow | 0.91 (0.52-1.59) | 0.50 (0.25-1.01) | 1.92 (1.06-3.47) | 1.24 (0.64-2.40) | 0.99 (0.58-1.70) | 0.63 (0.34-1.17) | 1.56 (0.96-2.53) | 1.28 (0.69-2.36) | 1.00 (0.59-1.70) | 0.81 (0.46-1.44) |
| Never married | 1.78 (1.31-2.43) | 0.89 (0.58-1.36) | 2.14 (1.61-2.82) | 1.18 (0.80-1.74) | 1.37 (1.04-1.81) | 0.62 (0.42-0.92) | 1.87 (1.48-2.38) | 0.92 (0.66-1.31) | 1.78 (1.40-2.28) | 0.93 (0.65-1.33) |
| **Wealth quintile** | | | | | | | | | | |
| 1 (lowest) | 1.00 | 1.00 | 1.00 | 1.00 | 1.00 | 1.00 | 1.00 | 1.00 | 1.00 | 1.00 |
| 2 | 1.44 (0.98-2.12) | 1.58 (1.04-2.42) | 1.26 (0.87-1.81) | 1.31 (0.90-1.92) | 0.66 (0.45-0.97) | 0.67 (0.44-1.01) | 0.84 (0.55-1.28) | 0.93 (0.61-1.44) | 1.16 (0.72-1.89) | 1.12 (0.67-1.88) |
| 3 | 1.70 (1.14-2.52) | 1.57 (1.01-2.45) | 2.00 (1.36-2.93) | 1.73 (1.15-2.61) | 0.85 (0.58-1.25) | 0.74 (0.48-1.13) | 1.48 (0.99-2.23) | 1.37 (0.88-2.12) | 1.69 (1.06-2.71) | 1.51 (0.91-2.49) |
| 4 | 3.92 (2.56-6.00) | 1.55 (0.91-2.66) | 3.48 (2.34-5.17) | 1.74 (1.10-2.75) | 1.33 (0.88-1.99) | 0.61 (0.37-1.00) | 2.72 (1.85-3.99) | 1.97 (1.27-3.07) | 2.30 (1.47-3.60) | 1.47 (0.85-2.54) |
| 5 (highest) | 18.48 (9.82-34.76) | 3.79 (1.61-8.90) | 7.96 (5.24-12.09) | 2.00 (1.18-3.40) | 4.55 (2.87-7.21) | 1.15 (0.61-2.17) | 5.75 (3.93-8.43) | 4.29 (2.45-7.50) | 6.03 (3.92-9.27) | 2.61 (1.41-4.85) |
| **Rurality** | | | | | | | | | | |
| Urban | 1.00 | 1.00 | 1.00 | 1.00 | 1.00 | 1.00 | 1.00 | 1.00 | 1.00 | 1.00 |
| Rural | 0.13 (0.09-0.20) | 0.45 (0.28-0.73) | 0.24 (0.18-0.32) | 2.25 (1.56-3.23) | 0.27 (0.20-0.36) | 1.93 (1.34-2.78) | 0.47 (0.37-0.59) | 0.78 (0.55-1.11) | 0.39 (0.31-0.50) | 1.19 (0.83-1.71) |
| **Reuse menstrual materials** | | | | | | | | | | |
| Yes | 0.33 (0.24-0.46) | 0.79 (0.53-1.18) | 0.38 (0.29-0.49) | 0.85 (0.62-1.16) | 0.48 (0.36-0.63) | 0.91 (0.65-1.26) | 0.31 (0.25-0.39) | 0.54 (0.41-0.71) | 0.35 (0.28-0.45) | 0.77 (0.58-1.02) |
| No | 1.00 | 1.00 | 1.00 | 1.00 | 1.00 | 1.00 | 1.00 | 1.00 | 1.00 | 1.00 |

OR: bivariate odds ratio. 95%CI: 95% confidence interval. aOR: odds ratio for full multivariable model including all listed predictors.
